# Helios Is Associated with CD4 T Cells Differentiating to T Helper 2 and Follicular Helper T Cells In Vivo Independently of Foxp3 Expression

**DOI:** 10.1371/journal.pone.0020731

**Published:** 2011-06-03

**Authors:** Karine Serre, Cécile Bénézech, Guillaume Desanti, Saeeda Bobat, Kai-Michael Toellner, Roger Bird, Susan Chan, Philippe Kastner, Adam F. Cunningham, Ian C. M. MacLennan, Elodie Mohr

**Affiliations:** 1 School of Immunity and Infection, MRC Centre for Immune Regulation, Institute for Biomedical Research, University of Birmingham, Birmingham, England, United Kingdom; 2 Institut de Génétique et de Biologie Moléculaire et Cellulaire, INSERM Unité 964, Centre National de la Recherche Scientifique, Unité Mixte de Recherche 7104, Université de Strasbourg, Strasbourg, France; Centre de Recherche Public de la Santé (CRP-Santé), Luxembourg

## Abstract

**Background:**

Although in vitro IL-4 directs CD4 T cells to produce T helper 2 (Th2)-cytokines, these cytokines can be induced in vivo in the absence of IL-4-signalling. Thus, mechanism(s), different from the in vitro pathway for Th2-induction, contribute to in vivo Th2-differentiation. The pathway for in vivo IL-4-independent Th2-differentiation has yet to be characterized.

**Findings:**

Helios (ikzf2), a member of the Ikaros transcription regulator family, is expressed in thymocytes and some antigen-matured T cells as well as in regulatory T cells. It has been proposed that Helios is a specific marker for thymus-derived regulatory T cells. Here, we show that mouse ovalbumin-specific CD4 (OTII) cells responding to alum-precipitated ovalbumin (alumOVA) upregulate Th2 features - GATA-3 and IL-4 - as well as Helios mRNA and protein. Helios is also upregulated in follicular helper T (TFh) cells in this response. By contrast, OTII cells responding to the Th1 antigen - live attenuated ovalbumin-expressing *Salmonella* - upregulate Th1 features - T-bet and IFN-γ - but not Helios. In addition, CD4 T cells induced to produce Th2 cytokines in vitro do not express Helios. The kinetics of Helios mRNA and protein induction mirrors that of GATA-3. The induction of IL-4, IL-13 and CXCR5 by alumOVA requires NF-κB1 and this is also needed for Helios upregulation. Importantly, Helios is induced in Th2 and TFh cells without parallel upregulation of Foxp3. These findings suggested a key role for Helios in Th2 and TFh development in response to alum-protein vaccines. We tested this possibility using Helios-deficient OTII cells and found this deficiency had no discernable impact on Th2 and TFh differentiation in response to alumOVA.

**Conclusions:**

Helios is selectively upregulated in CD4 T cells during Th2 and TFh responses to alum-protein vaccines in vivo, but the functional significance of this upregulation remains uncertain.

## Introduction

CD4 T helper 2 (Th2) cells are protective when they control immunity to extracellular parasites but their involvement in allergic inflammatory responses shows they can also be pathogenic. The canonical Th2-cytokine is IL-4 which is often produced with IL-5 and IL-13 and the genes encoding these 3 cytokines are located in a contiguous gene cluster [1]–[3]. There are still gaps in our understanding of how CD4 Th2 cells are induced *in vivo* [4]. To get insight into the specific features and the signalling pathway(s) that operate(s) *in vivo* we studied Th2-differentiation of naïve ovalbumin (OVA)-specific transgenic CD4 T (OTII) cells that had been transferred into wild-type congenic mice. Alum-precipitated ovalbumin (alumOVA) was used to induce the Th2-associated transcription factor GATA-3 and IL-4, while live attenuated ovalbumin-expressing *Salmonella* (SalOVA) was used to induce Th1-associated T-bet and IFN-γ. a previous report from our laboratory extensive analysis of the diversity of these different CD4 T cell responses was made by using low-density, real-time RT-PCR microfluidic cards [5]. One of the novel outcomes of this study was that, by 3 days after immunization, the transcription regulator Helios was induced in OTII cells in response to alumOVA but, not to SalOVA. The objective of the current report is to assess whether this selective expression of Helios is strictly associated with Th2 cells differentiated *in vivo*.

Helios (Ikzf2) belongs to the Ikaros transcription factor family that is made up of five DNA-binding proteins; Ikaros, Helios, Aiolos, Eos and Pegasus [6]. These transcription factors contain two sets of highly conserved C2H2 zinc fingers motifs. At the N terminus, four zinc fingers are responsible for sequence-specific DNA binding, while at the C terminus two zinc fingers enable homodimeric or heterodimeric interactions between family members [7], [8]. Ikaros, Helios and Aiolos expression is restricted to cells of the hematopoietic system, whereas Eos and Pegasus are more widely expressed [9], [10]. Ikaros and Aiolos are involved in many aspects of B cell differentiation and functions [11]–[17]. Ikaros also appears to be important during T cell development, and its absence leads to increased double-negative, stage 4, thymocyte proliferation [18], [19]. In mature T cells, Ikaros has been reported both to regulate Th2 commitment and silence Th1 differentiation [20]–[22]. In addition, Eos mediates gene silencing in regulatory T cells (Tregs) in a Foxp3-dependent manner [23]. Finally, Helios is highly expressed at early stages of thymocytes development [9], [24]. In mature T cells, Helios expression has been strongly associated with Tregs by several groups [25]–[30]. Helios has also been observed in a very small number of non-characterized splenic germinal centers cells [9]. By dimerizing with Ikaros, Helios potentially controls its epigenetic function by altering the intracellular localization of Ikaros [9], [24], [31]. This suggests a possible role for Helios in the acquisition of selective functions by peripheral CD4 T cells.

Helios expression has also been associated with naturally occurring regulatory T cells [27], but we show that Helios upregulation in the context of the response to alumOVA occurs independently of Foxp3 expression. Finally, we generated Helios-deficient OTII cells and used these to probe the role of Helios in the acquisition of Th2-features in response to alumOVA.

## Results

### Helios is selectively expressed in CD4 T cells committing to Th2 differentiation

**Figure 1:**
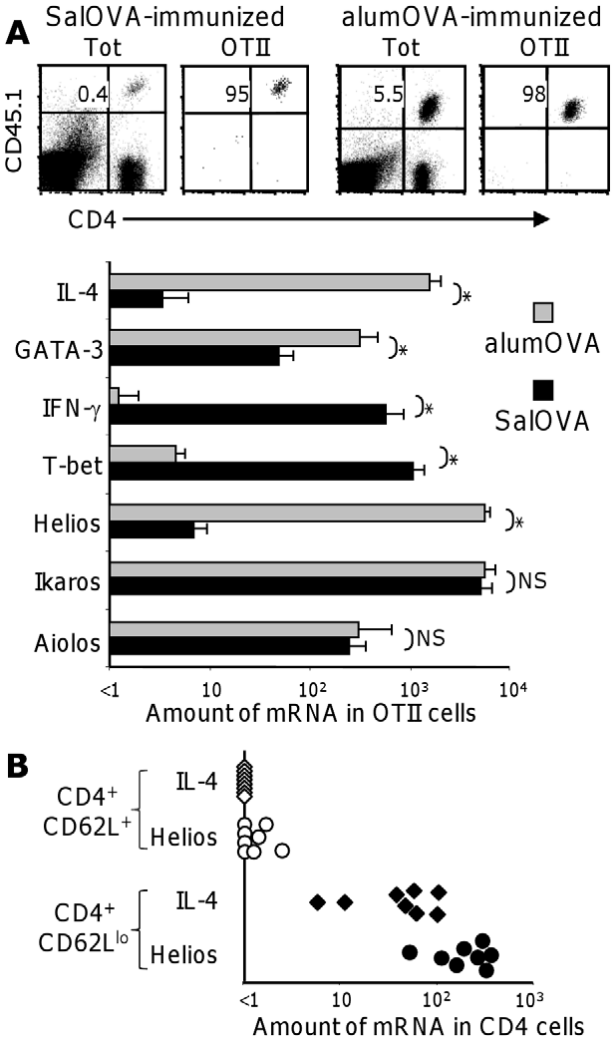
Helios mirrors IL-4 mRNA expression in CD4 T cells responding to Th2-antigens in vivo. **A**) One day after receiving CD45.1^+^ OTII cells groups of congenic CD45.2^+^ B6 mice were immunized either with SalOVA or alumOVA in both footpads. Seven days later popliteal LN cells were taken and OTII cells were sorted on the basis of CD4 and CD45.1 expression. The right hand graphs marked OTII show the purity of the sorted cells, which were used to prepare cDNA. The bar charts show IL-4, GATA-3, IFN-γ, T-bet, Helios, Ikaros and Aiolos mRNA levels +SD, assessed by real-time PCR relative to the level of β2-microglobulin mRNA. The data are representative of 2 independent experiments with a total of 4 mice. The Mann-Whitney 2-tailed test was used to estimate the significance of differences between groups: * = p<0.05, NS = not significant. **B**) C57BL/6 mice were immunized with the flagellin subunit FliC intraperitoneally. Seven days later CD4 T splenocytes were FACS-sorted as naïve (CD62L^+^) and primed (CD62L^lo^) cells. The relative amounts of IL-4 and Helios mRNA were determined by real-time RT-PCR relative to the level of β2-microglobulin mRNA. The data are representative of 2 independent experiments with 8 mice in total.

Our previous studies show that CD4 OTII cells primed to SalOVA or alumOVA respectively acquire Th1 and Th2 features as early as 3 days after immunization. These studies also suggest that Helios expression is selectively associated with Th2 differentiation [5]. To confirm these findings, chimeric mice were constructed by transfer of CFSE-labelled CD45.1^+^ OTII cells into congenic wild-type CD45.2^+^ recipients. The day after cell transfer chimeras were immunized in both footpads, with either SalOVA or alumOVA and 7 days after immunization OTII cells were FACS-sorted from the draining popliteal lymph node (LN) (Fig. 1A). Real-time RT-PCR shows the sorted OTII CD4 T cells responding to alumOVA selectively express high levels of IL-4, GATA-3 and Helios mRNA (Fig. 1A). By contrast, OTII cells responding to SalOVA acquire Th1 features, expressing high levels of IFN-γ and T-bet but not Helios mRNA (Fig. 1A). Importantly, SalOVA- and alumOVA-primed OTII cells express both Ikaros and Aiolos mRNA to similar levels. Thus, Helios mRNA is induced in OTII cells responding to alumOVA but not to SalOVA (Fig. 1A and [5]).

We next tested whether the selective association of IL-4 with Helios expression occurs in response to another antigen that induces differentiation of naïve CD4 T cells to Th2 cells. To this end we immunized mice with FliC, a subunit of the *Salmonella* flagellar protein – flagellin. In addition to being the target for specific immune recognition by T and B cells, FliC is a ligand for TLR-5 and it induces a strong Th2 response [32], [33]. WT mice were immunized intraperitoneally with FliC and 7 days later high levels of IL-4 and Helios mRNA were found in FACS-sorted CD4^+^CD62L^lo^ (primed) splenocytes (Fig. 1B). By contrast these mRNAs were not present in the CD4^+^CD62L^+^ (naïve) population. Thus, Helios mRNA is upregulated in splenic and LN CD4 T cells responding to at least two types of Th2 antigens.

### Kinetics of Helios expression in OTII cells responding to alumOVA mirrors GATA-3 expression

**Figure 2:**
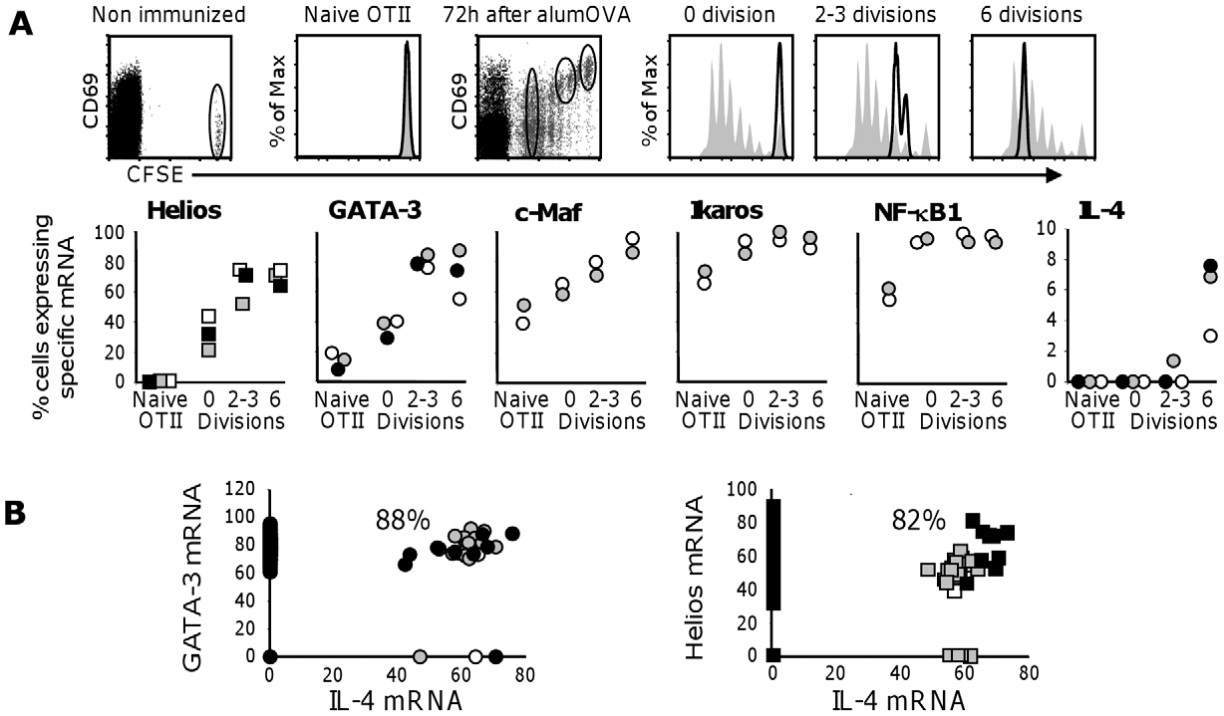
Transcription factor mRNA related to IL-4 induction in single alumOVA-responding OTII cells. C57BL/6 mice received CFSE-labelled OTII cells and were then immunized with alumOVA in both footpads. Three days later flow cytometry was used to analyze and sort single cells from the draining LN. **A**) The dot plots in the top row are gated on CD4 T cells and show the CD69 expression as a function of OTII cell proliferation. Naïve LN cells from a group of non-immunized chimeras were used as controls. Histograms show the populations of single OTII cells from popliteal LN isolated as follows: 1) naïve OTII cells, 2) OTII cells that had not divided, but were CD69^+^ (0 division), 3) OTII cells that had completed 2–3 divisions, 4) OTII cells that had completed 6 divisions. Histograms show the purity of the sorted OTII cells (black lines) within the initial OTII population (filled grey). The lower set of graphs show the percent of single cells expressing β2-microglobulin mRNA that also express IL-4 or transcription factor mRNA as quantified using a duplex RT-PCR in the populations of naïve and responding OTII cells defined above. β2-microglobulin expression indicated more than 90% of 154 wells from each sorted population contained a cell. **B**) Percent of single OTII cells that had divided 6 times that expressed β2-microglobulin mRNA simultaneously with either GATA-3 (left panel) or Helios (right panel) as quantified by a triplex RT-PCR. The results in A & B are shown for 2 to 3 independent experiments respectively represented by open, grey and black symbols.

We next set out to identify the proportion of OTII cells that upregulate Helios at successive stages in the response to alumOVA. Again CFSE-labelled OTII cells were transferred into congenic wild-type mice and these chimeras were immunized in the footpads with alumOVA. Three days after immunization single OTII cells were FACS-sorted from the popliteal LN as a function of CD69 expression and the number of cell division accomplished (Fig. 2A). In this way individual cells at different stages of the response were obtained: (i) cells that had upregulated CD69, but had not yet started to divide (termed 0 division in Fig. 2); (ii) cells that had divided 2–3 times based on CFSE dilution (2–3 divisions); and (iii) cells that had divided 6 times (6 divisions). Naïve single OTII cells from the popliteal LN of chimeras that had not been immunized were also studied. Levels of mRNA for IL-4 and related transcription factors were then determined by real-time RT-PCR in the single cells derived from each of these 4 OTII subsets (Fig. 2A). The transcriptional regulators involved in Th2 development that we analyzed were – GATA-3 [34], [35], c-Maf [36]–[39], Ikaros [20], [22], and NF-κB1 [40]–[42].

**Figure 3:**
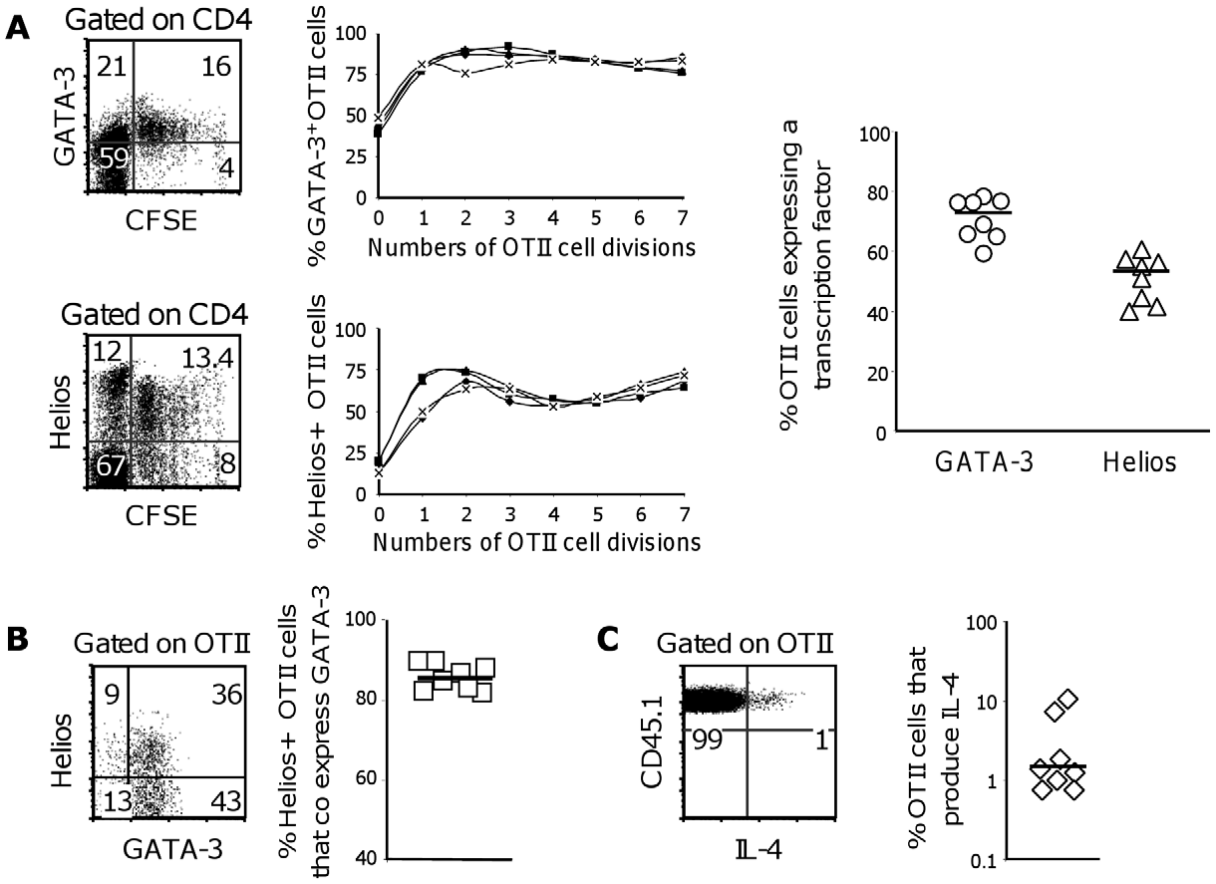
Transcription factor and IL-4 protein expression in single alumOVA-responding OTII cells. C57BL/6 mice received CFSE-labelled, or not, OTII cells and were then immunized with alumOVA in both footpads. Three days later flow cytometry was used to analyze the expression of transcription factor and IL-4 proteins in OTII cells from the draining LN. **A**) Dot plots gated on CD4 T cells show GATA-3 and Helios protein expression in the draining LN suspensions as a factor of CFSE dilution. Cells to the left of the vertical quadrant bar are CFSE^−^ endogenous CD4 T cells. Histograms on the right of each dot plot show the proportions of OTII cells that had completed the indicated number of divisions and express either GATA-3 (top) and Helios (bottom). Each line represents results from one mouse. Data are representative of 2 independent experiments. The graph on the right hand shows the proportion of OTII cells from each of 8 mice expressing GATA-3 or Helios. **B**) The dot plot shows the co-expression of Helios and GATA-3 proteins by OTII cells. The graph on the right shows that more than 80% of the OTII cells from each of 8 mice that are positive for Helios also express GATA-3. **C**) The proportion of IL-4-producing cells within the OTII cells after 5 h restimulation *in vitro* with OVA-peptide was assessed by intracellular flow cytometry as shown in the dot plot. The graph shows the proportions of cells in the responding LN from 8 mice that expressed IL-4. The data in **B–C**) are representative of 3 independent experiments.

Helios mRNA, in contrast to the other transcription factors, is expressed in fewer than 2% of the naïve OTII cells, but is already induced in 30% of CD69^+^ OTII cells that have not yet divided (0 division) (Fig. 2A). The proportion of cells expressing Helios then increases linearly through to 6 cell divisions. GATA-3 mRNA is present in approximately 20% of naïve OTII cells (Fig. 2A). This proportion rises to 40% in the CD69^+^ OTII cells that had not divided. Some 80% of primed OTII cells that have completed 2–3 divisions express GATA-3, and this expression is maintained through 6 divisions. The profile of c-Maf expression is similar although this transcription regulator is present in a higher proportion of naïve cells (40%). Ikaros and NF-κB1 are constitutively expressed in 60–70% of naïve OTII cells and this is rapidly up-regulated in the remaining OTII cells following immunization. As previously reported IL-4 mRNA expression is not seen prior to cell division [43], but becomes apparent in some 7% of the OTII cells that have undergone 6 divisions. A similar proportion of IL-4-secreting OTII cells express intracellular IL-4 protein at this stage (see Refs [44]–[46] and Fig. 3C). Helios and GATA-3 mRNA expression were respectively seen in 88% and 82% of the OTII cells induced to express IL-4 mRNA (Fig. 2B). We were unable to determine if the few IL-4-producing cells that did not contain Helios or GATA-3 had expressed these transcription factors earlier in the response.

We went on to assess whether the proportion of OTII cells expressing Helios and GATA-3 mRNA was related to the proportion of cells expressing these transcription factors at the protein level (Fig. 3). The proportion of OTII cells with GATA-3 and Helios proteins is shown in relation to the number of cell divisions induced by alumOVA in Fig. 3A. This shows that kinetics of upregulation of both of these proteins parallels the upregulation of their mRNA, as shown in Fig. 2A. In addition, more than 80% of Helios-expressing OTII cells also express GATA-3 (Fig. 3B). Overall these results show similar timing and frequency of Helios and GATA-3 mRNA and protein expression during this Th2 response.

### OTII cells induced to express Helios by alumOVA do not express Foxp3

**Figure 4:**
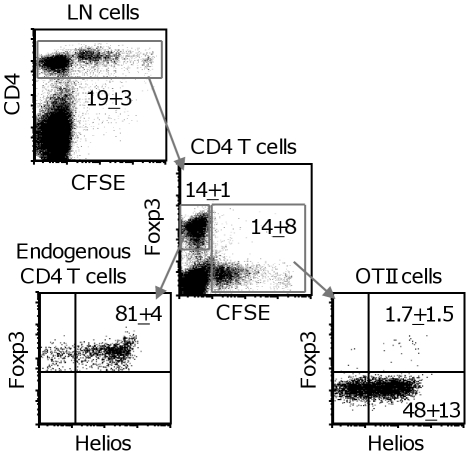
OTII cells induced to express Helios by alumOVA do not express Foxp3. C57BL/6 mice received CFSE-labelled OTII cells and were then immunized with alumOVA in the footpads. Three days later the draining LN were taken for analysis by flow cytometry of CFSE content and the expression of Helios, and Foxp3 on endogenous CD4 T cells and OTII cells. The top left dot plot shows the expression of CD4 and CFSE and its resolution into the central dot plot with Foxp3 and CFSE content of cells in CD4 T cell gate. The bottom left dot plot shows Foxp3 and Helios by Foxp3+ endogenous CD4 T cells that contain no CFSE. The bottom right dot plot shows Foxp3 and Helios expression by OTII cells captured in the CFSE+ CD4+ gate. The median percentages and SD for the mice studied (2 independent experiments n = 8 in total) are shown in each dot plot.

Helios expression has been associated with naturally occurring regulatory T cells [27]. For this reason we went on to assess if the OTII cells that have been induced to express Helios by alumOVA also express Foxp3. Again the LN response of CFSE-labelled OTII cells was evaluated. OTII cells were identified by their CFSE content as CD4^+^ CFSE^+^ cells (Fig. 4). Three days after chimeras were immunized with alumOVA a mean of 48%±13 of the OTII cells in the draining LN contained Helios. Within the OTII population very few (1.7%±1.5) co-expressed Helios and Foxp3. Thus, the majority of the Helios^+^OTII cells are Foxp3^−^. As reported in Ref [27], 84%±4 of the 14%±1 of endogenous CD4 T cells that expressed Foxp3 are also Helios positive. These data show that almost all of OTII cells responding to alumOVA that are induced to express Helios do so in a different manner from that of naturally occurring regulatory T cells.

### Helios is induced in both TFh cells and other CD4 effectors responding to alumOVA

Upon immunization CD4 T cells first encounter antigen presented on the surface of dendritic cells in the T zone near high endothelial venules (HEV). By 72 h the expression of CXCR5 enables a proportion of the responding CD4 T cells to migrate into the B follicles [44], [46]. These migrant cells are TFh required for the selection of B cells in germinal centers (GC) and for inducing the selected B cells to differentiate into plasma cells, memory B cells or centroblasts [47]. CXCR5^high^CCR7^low^ T cells have been found to have elevated IL-4 and PD-1 transcript expression [48] and GC are the focus of IL-4 production during Th2-responses [49]–[51].

**Figure 5:**
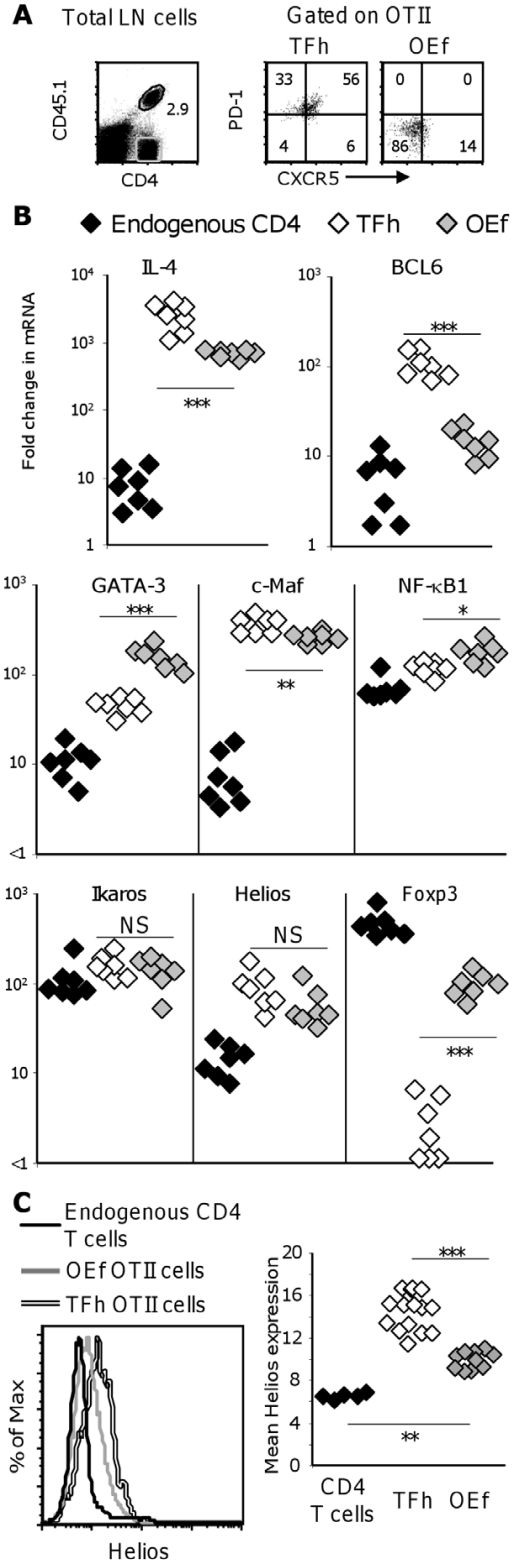
Helios is induced in both T zone effector and follicular helper OTII cells. **A**) Popliteal LN cell suspensions were prepared from OTII cell chimeras immunized 7 days previously with alumOVA in both footpads. The left hand FACS plot shows the CD4+CD45.1+ OTII cells (black gate) and the largely non-responding endogenous CD4+CD45.1− T cells (grey gate). The OTII cells were sorted (right hand pair of dot plots) into TFh cells (CXCR5+PD-1+) and other effector cells OEf (CXCR5−PD-1−). **B**) The relative amounts of mRNA for IL-4 and a range of transcription factors was determined by real-time RT-PCR. The values for cells from the responding LN from 8 mice in 3 experiments is shown in the graphs: Endogenous CD4 T cells (black diamonds), OEf (grey diamonds), TFh (open diamonds). **C**) The histogram shows the level of expression of Helios protein in the TFh and OEf from a responding LN, sorted as in (A), compared with that by endogenous FOXP3- CD4 T cells from the non-responding brachial of the same mouse (sorted using gates shown in Fig 4). The graph shows the geometric mean expressions in the same three populations in the LN from each of 8 mice in 3 experiments. The symbols are as in (B). Mann-Whitney 2-tailed statistical probabilities of differences between the TFh and OEf OTII groups are indicated: NS = not significant, * = p<0.05, ** = p<0.01, *** = p<0.001.

To test if Helios is expressed in TFh cells, seven days after immunizing a further set of chimeras with alumOVA PD-1^+^CXCR5^+^ TFh cells and PD-1^−^CXCR5^−^ other effector (OEf) cells were sorted from the draining LN (Fig. 5A). Largely non-responding endogenous CD45.1^−^ CD4 T cells were also sorted as controls. Real-time RT-PCR shows that both TFh and OEf OTII cells significantly upregulate IL-4 at the population level compared to endogenous CD4 T cells. Nevertheless, TFh OTII cells produce significantly more IL-4 mRNA than in the OEf OTII cells (Fig. 5B). Changes in the expression of transcription factors associated with TFh or Th2 differentiation or both of these are also shown in Fig. 5B. As expected, OEf OTII cells were found to contain significantly more GATA-3 mRNA than TFh OTII cells, and TFh OTII cells strongly express BCL6 mRNA whereas OEf OTII cells do not. There are no major differences in c-Maf, NF-κB1, and Ikaros between TFh and OEf OTII cells. Finally, as expected, Foxp3 is expressed at much lower levels in the responding OTII cells than in endogenous CD4 T cells.

Both TFh and OEf OTII cells have significantly more Helios mRNA than the endogenous CD4 T cells, but the trend for TFh to express more Helios than OEf was not significant. To clarify this, we have compared Helios protein expression in OEf and TFh OTII cells with that in non-responding endogenous CD4 T cells excluding regulatory T cells as they express high level of Helios (Fig. 5C). This shows that TFh OTII cells reproducibly and significantly express higher level of Helios than OEf and these in turn express more Helios than the non-responding CD4 T cells other than regulatory T cells.

### Helios, like IL-4 and IL-13, requires NF-κB1 for its induction in CD4 T cells by alumOVA

**Figure 6:**
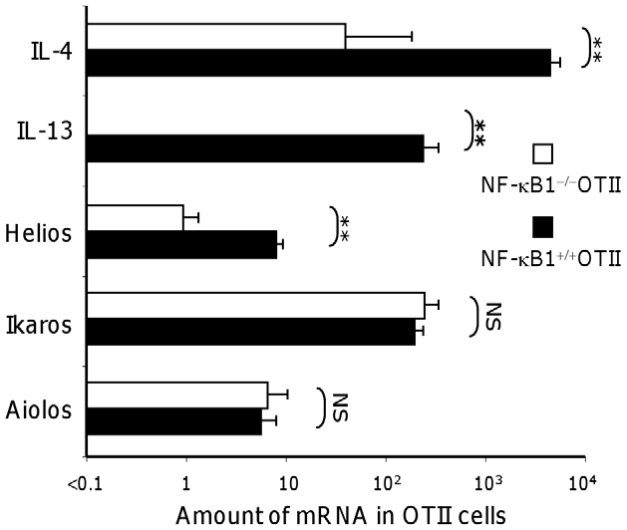
Helios and Th2 cytokine mRNA induction in CD4 T cells by alumOVA requires NF-κB1. C57BL/6 mice received CFSE-labeled NF-κB1^+/+^OTII cells or NF-κB1^−/−^OTII cells and were then immunized with alumOVA in both footpads. Three days later *in vivo*-primed NF-κB1^+/+^ (black bars) or NF-κB1^−/−^ (open bars) OTII cells were FACS-sorted as CFSE^+^ CD4 T cells. The relative Th2-cytokine and Ikaros family of transcription factor mRNA levels were determined by real-time RT-PCR relative to β2-microglobulin mRNA expression. The data are derived from 2 independent experiments with a total of 6 mice per group. Mann-Whitney 2-tailed statistical probabilities of differences between groups are indicated: NS = non significant, ** = p<0.01.

NF-κB1-deficient mice are impaired in Th2 responses including experimental allergic airway inflammation and intestinal helminth infection [40]–[42], [52], [53]. In addition, we have recently published evidence that the induction of IL-4, IL-13 and CXCR5 by alum-protein vaccine is under NF-κB1 control [54]. On the other hand, NF-κB1-deficient CD4 T cells still become effectors that upregulate IL-2, IL-21 and IFN-γ mRNA and provide help for extrafollicular antibody responses. To test if NF-κB1-deficiency in CD4 T cells selectively affects Helios upregulation, chimeras were constructed by transferring either NF-κB1^+/+^OTII cells or NF-κB1^−/−^OTII cells into wild-type congenic recipients. Three days after footpad immunization with alumOVA NF-κB1^+/+^OTII cells from the draining node expressed 100 fold more IL-4 mRNA and at least 1000 fold more IL-13 mRNA than NF-κB1^−/−^OTII cells (Fig. 6). NF-κB1^+/+^OTII cells expressed 10 fold more Helios mRNA than NF-κB1^−/−^OTII cells, while both Ikaros and Aiolos were similarly expressed in the two cell types. Thus, optimal Helios expression, like that of IL-4 and IL-13, requires NF-κB1-signaling.

### Helios is not expressed during in vitro IL-4 directs CD4 Th2 cell polarization

**Figure 7:**
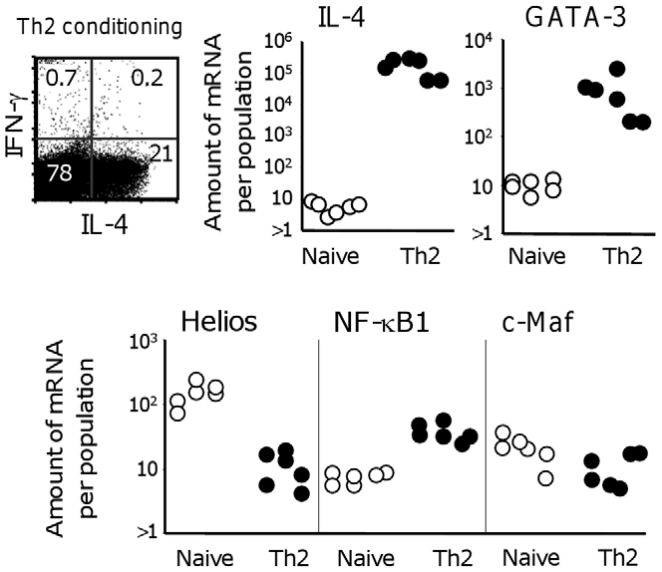
Helios is not induced during IL-4-directed in vitro polarization of CD4 T cells. LN cell suspensions from OTII mice were cultured in standard conditions for 6 days to induce IL-4-producing cells *in vitro*. The cultures included OVA peptide+IL-4+neutralizing anti-IL-12 and anti-IFN-γ. Flow cytometry dot plots show cells producing IL-4 versus IFN-γ. The graphs show expression of IL-4, GATA-3, Helios, NF-κB1 and c-Maf mRNA relative to β2-microglobulin mRNA by freshly-isolated total LN cell suspensions (open circles), or by cells after Th2 differentiation (black circles). Data show duplicate culture results from 3 independent experiments.

Although, there are key molecules in CD4 T cells that are upregulated and are involved in the induction of Th2 cytokine *in vivo*, this does not necessarily imply that they are required in the *in vitro* Th2-conditionned culture system. We carried out further experiments to assess whether Th2-transcription factors are induced when CD4 T cells are induced to produce Th2 cytokines by being activated through their TCR in the presence of IL-4 *in vitro*. As expected this treatment strongly upregulated GATA-3 and IL-4 in the cultured cells (Fig. 7). By contrast neither Helios nor c-MAF are induced, while NF-κB1 is modestly upregulated. These findings further highlight the differences between *in vitro* and *in vivo* responses that induce Th2 cytokines. The lack of Helios upregulation in the IL-4-directed *in vitro* Th2-polarization may explain why Helios^−/−^CD4 T cells secrete Th2 cytokines, similarly than WT CD4 T cells, when polarized in IL-4-directed *in vitro* stimulation [55].

### Helios is not required for Th2 or TFh development in responses to alumOVA

The data presented so far suggest Helios is a candidate transcriptional regulator of Th2 and TFh differentiation *in vivo* at least in response to alum-precipitated protein. To test if Helios is required for the differentiation of CD4 T cells we next set up studies to compare Th2 and TFh differentiation and function induced by alumOVA in Helios^+/+^ or Helios^−/−^OTII cells. These were transferred into wild type congenic mice and again their response to footpad immunization with alumOVA was followed.

Helios^−/−^ mice have been described elsewhere [55], briefly in this mutant strain Helios lacks most of exon 7, including the sequences that encode the C-terminal zinc finger domain that is essential for dimerization. This deletion results in loss of Helios protein. The homozygous Helios-deficient mice have a low survival rate and the mutants die during the first week. Exceptionally, when mice survive the critical first week, these mice have normal differentiation and homeostasis of αβ and γδ T cells, NK T cells and regulatory T cells. Because of the low survival of Helios^−/−^ pups [55], we crossed heterozygous Helios^+/−^ mice with OTII mice to generate Helios^+/−^OTII^+/−^ mice. These hybrids were then interbred and the resulting foetal liver cells prepared from day 14 Helios^−/−^OTII^+^ embryos were used to reconstitute lethally-irradiated congenic C57BL/6 mice and generate Helios^−/−^OTII cells. CD45.2^+^ LN cells from Helios^+/+^OTII^+^ or Helios^−/−^OTII^+^ chimeras were then prepared, CFSE-labelled and transferred into CD45.1^+^ congenic wild-type recipient mice.

**Figure 8:**
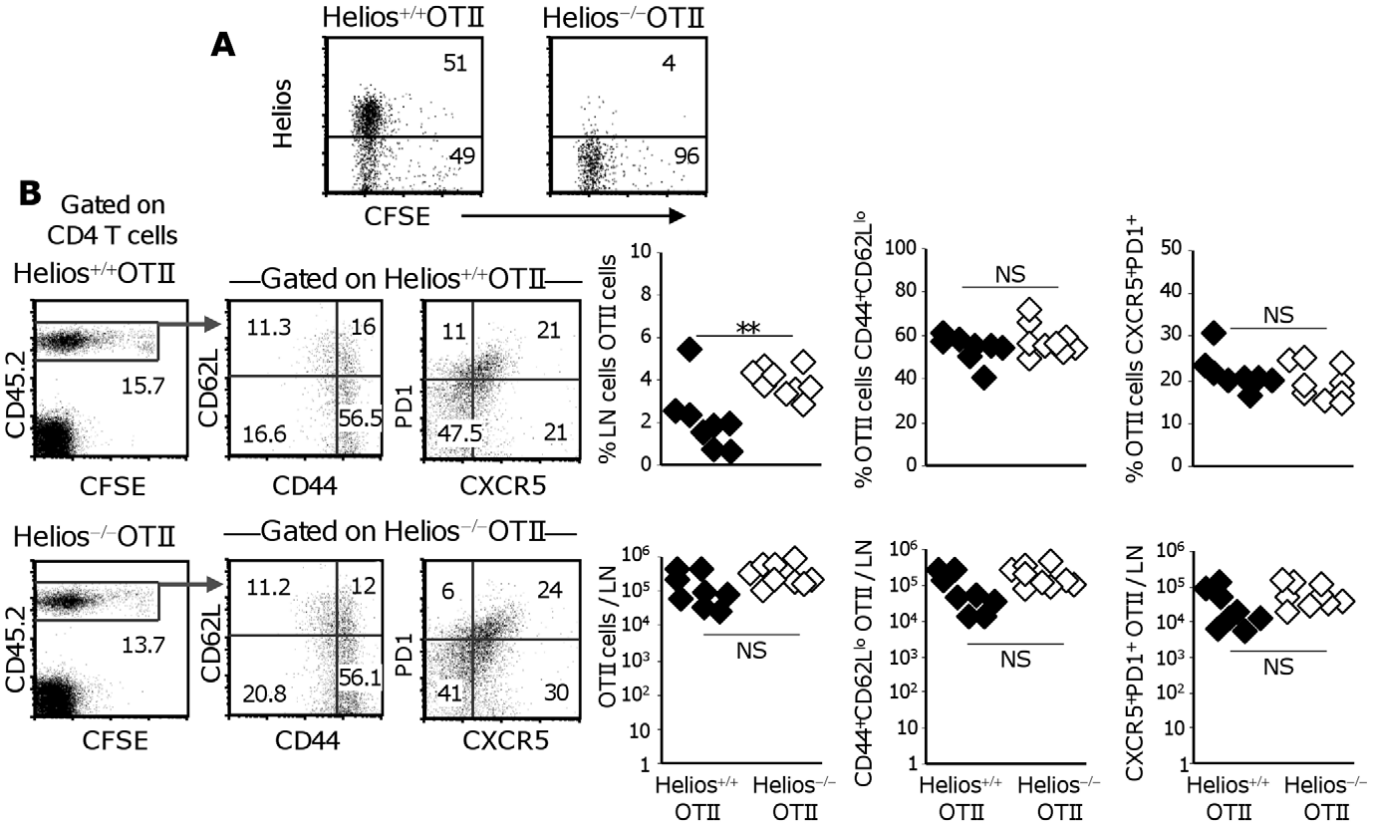
Helios is not required for Th2 or TFh cell induction in response to alumOVA in vivo. One day after receiving Helios^+/+^ or Helios^−/−^CD45.2+OTII cells congenic CD45.1^+^ B6 mice were immunized with alumOVA in both footpads. **A**) Seven days later the draining LN were taken for analysis by flow cytometry of CFSE content and the expression of Helios. **B**) The proportion of OTII cells in the CD4 T cells was assessed as CD4^+^ CD45.2^+^ cells (left hand dot plots). The middle and right hand dot plots are gated on OTII cells and show the proportions of effector cells, identified as CD4^+^CD45.2^+^CD44^+^CD62L^lo^ and TFh cells, identified as CD4^+^CD45.2^+^CXCR5^+^PD-1^+^ cells. The graphs show the percentages (top) or total numbers (bottom) of cells in LN. Left graphs compare all Helios^+/+^ OTII cells (black diamonds) with all Helios^−/−^ OTII cells (open diamonds). The centre graphs compare those cells in these populations that are CD44^+^CD62L^lo^ OTII effectors. The right graphs compare the CXCR5^+^PD-1^+^ TFh cells (right). The data are derived from 2 independent experiments with 8–9 mice in total. Statistical differences between groups are indicated and NS = not significant, ** = p<0.01.

Seven days after immunization with alumOVA no Helios protein was detected by intracellular flow cytometry in the Helios^−/−^OTII cells from the draining LN while about 50% of the Helios^+/+^OTII cells express this transcription regulator (Fig. 8A). The extent of proliferation and survival by Helios^+/+^ and Helios^−/−^OTII cells was comparable when assessed 7 days after immunization (Fig. 8A&B). Helios-deficiency in OTII cells did not alter the proportions and total numbers of effector CD62L^lo^CD44^+^ OTII cells, or theCXCR5^+^PD-1^+^ TFh OTII cells generated (Fig. 8B).

### Helios deficiency in CD4 T cells does not modify their capacity to provide help for follicular or extrafollicular antibody response to alumOVA

**Figure 9:**
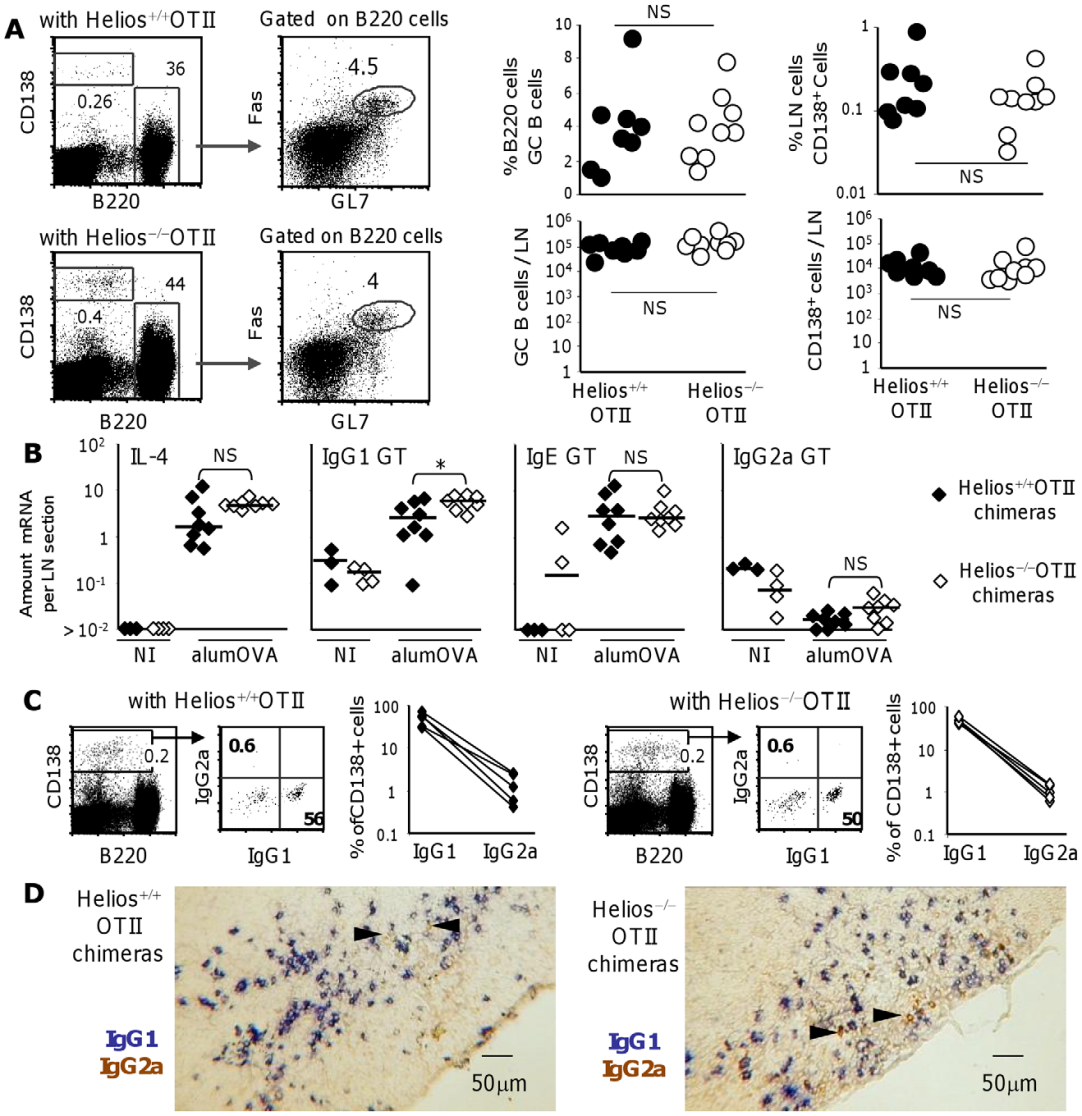
Helios^−/−^ OTII cells responding to alumOVA induce IgG class switched plasma cells at comparable levels to those of Helios^+/+^ OTII cells. Chimeras were constructed by transfer of Helios+/+ or Helios−/−CD45.2+OTII cells into congenic CD45.1^+^ B6 mice. One day later they were immunized with alumOVA in both footpads and the B cell response in the draining LN at 7 days was assessed. **A**) Representative flow cytometry plots show gating of antibody-producing cells (B220^int^CD138^+^) and GC B cells (B220^+^Fas^+^GL7^+^). The graphs show the percentages (top) or the numbers (bottom) of GC B cells (left) or antibody-producing cells (right) in the two groups of chimeras. The data are derived from 2 independent experiments with 8–9 mice in each group. **B**) The graphs compare the levels of IL-4 mRNA and Cγ1, Cε and Cγ2a germline transcripts (GT) per LN section in each of the 8 chimeras in both groups. **C**) Dot plots show the gating of IgG1 and IgG2a, B220+CD138+ antibody-producing cells. The graphs show the % B220^+^CD138^+^ cells that were IgG1 expressing or IgG2a expressing in the draining LN of each of 5 chimeras in the two groups. **D**) Photomicrographs of sections of a draining LN from a representative Helios^+/+^OTII cell chimera (left) and Helios^−/−^OTII cell chimera (right). IgG1^+^ cells are blue, IgG2a^+^ are brown. Black arrow heads highlight rare IgG2a-producing cells. All results are from 2 independent experiments. The Mann-Whitney 2 tailed statistical differences between groups shown are indicated and NS = not significant, * = p<0.05, ** = p<0.01.

We next set out to test the effect of Helios deficiency in antigen-specific CD4 T cells on their capacity to support antibody responses to alumOVA. The B cell response induced by this Th2-inducing antigen is characterized by switching to IgG1 [32], [56]–[60]. Alum-protein antigens injected subcutaneously selectively induce processed γ1 and ε Ig heavy chain gene germline transcripts in the draining LN. Productive switching to IgG1 occurs in these responding nodes although there is very little switching to IgE in these sites [32], [57], [58]. Non-immunized popliteal LN have few if any plasma cells or GC, so the appearance of these features after immunization is strong evidence of an induced response. In addition, T cell help is limiting in the primary response to alum-protein and by 7 day after immunization there are only modest numbers of plasma cells induced. By contrast, the transfer of 10^6^ OTII cells i.v. increases the 7 day plasma cell count by some 1000 fold and a high proportion of these plasma cells have switched to IgG1 [59], [60]. These considerations allowed us to assess the role of Helios in the induction of help for antibody responses by the transfer of Helios^+/+^ or Helios^−/−^OTII cells into wild type congenic mice and observing the popliteal LN response to alumOVA. The 7 day antibody response induced is shown in Fig. 9. The numbers of germinal centre cells (Fas^+^GL7^+^B220^+^) and plasmablasts and plasma cells (collectively CD138^+^) is comparable in the two sets of chimeras (Fig. 9A). It will be seen that the IL-4, γ1 and ε transcripts are all strongly induced in these LN (Fig. 9B). There is no difference in the levels of IL-4 and ε transcripts induced between the Helios^+/+^ or Helios^−/−^OTII cell chimeras. Paradoxically there is a borderline significant increase in the level of γ1 germline transcripts in the Helios^−/−^OTII cell chimeras. This finding was not pursued further as the proportions of plasma cells producing IgG1 were similar in the 2 sets of chimeras (Fig. 9C, D). There was no increase in the low level of switching to IgG2a, in the Helios^−/−^ chimeras. This indicates that there was no alteration of polarization from Th2 to Th1 as switching to this isotype is typically associated with Th1 responses (Fig. 9C, D).

### Helios does not regulate the induction of Th2 cytokine expression in CD4 T cells responding to alumOVA

**Figure 10:**
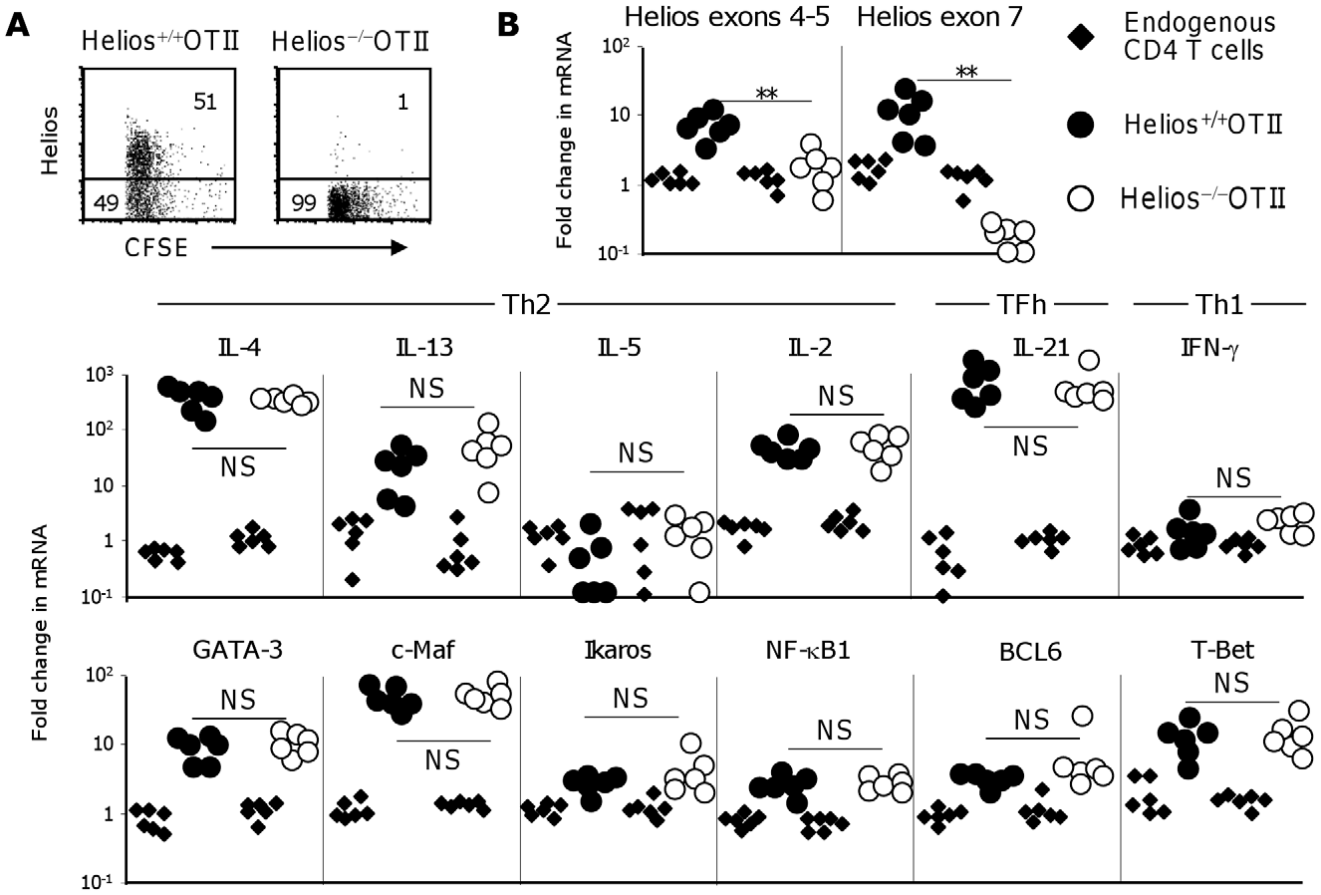
Induction of Th2/TFh-cytokines and transcription factors in CD4 T cells by alumOVA does not require Helios. One day after receiving Helios^+/+^ or Helios^−/−^ OTII cells recipient B6 mice were immunized with alumOVA in both footpads. **A**) Three days later the draining LN were taken for analysis by flow cytometry of CFSE content and the expression of Helios. **B**) At this stage the Helios^+/+^ (black circles) or Helios^−/−^ (open circles) OTII cells were FACS-sorted as CFSE^+^ CD4 T cells. Largely non-activated endogenous CFSE^−^ CD4 cells were used as controls (black diamonds). The relative mRNA levels of Th2, TFh and Th1 cytokines (top row) or transcription factors (bottom row) were determined by real time RT-PCR relative to the level of β2-microglobulin mRNA expression. Each symbol represents sorted cells pooled from the two popliteal LN of one mouse. The data are derived from 2 independent experiments with 6 mice in total. Statistical differences between groups are indicated and NS = not significant, ** = p<0.01.

Finally, we assessed the role of Helios in transferred OTII cells in the induction of cytokines and transcription factors in response to alumOVA *in vivo*. Again recipient mice received CFSE-labelled Helios^+/+^ or Helios^−/−^OTII LN cells and were immunized the next day with alumOVA in both footpads. In these experiments the responding OTII cells were evaluated 3 days later. As previously shown (Fig. 8A), the Helios^−/−^OTII cells do not express Helios protein (Fig. 10A). In addition, the Helios^+/+^ or Helios^−/−^OTII cells were FACS-sorted and assessed by real-time RT-PCR for Helios mRNA detected with primers recognizing either the junction between exons 4 and 5 or the deleted sequence of exon 7 (Fig. 10B). This shows that Helios mRNA is not induced in Helios^−/−^OTII cells. OTII cells expressed 1000 fold more IL-4 and 50 fold more IL-13 mRNA than the largely non-OVA-specific and hence non-responding endogenous CD4 T cells (Fig. 10B). Hence, the responses of the Helios^+/+^OTII cells are comparable to those described in previous sections. Additionally, no obvious difference was found between the responses of Helios^+/+^ and those of Helios^−/−^OTII cells in relation to the production and transcriptional control of Th2 or TFh cytokine production. Also, the absence of Helios does not lead to the induction of the Th1 features - IFN-γ or T-bet. These results indicate that Helios is dispensable for the regulation of both Th2 and TFh cytokines and transcription factors in response to alumOVA immunization.

## Discussion

In mature hematopoietic cells, Helios expression is restricted to T cells [9], [24], suggesting that Helios may control important aspects of T cell differentiation and/or function. Strikingly, the kinetics of Helios expression mirrors that of GATA-3 with about 60% of alumOVA-primed OTII cells expressing Helios after 2–3 divisions. This is shown both at the mRNA and protein levels. Other indirect evidence linking Helios with Th2 responses comes from a genome wide association study. This identified Helios in a sequence variant that correlates with high numbers of blood eosinophils in asthma [61]. Our data also show that Helios expression appears to be regulated by NF-κB1, a transcription factor associated with Th2 responses [40]–[42], [54].

We found that Helios is not induced in OTII cells responding to the Th1 antigen SalOVA, while Ikaros and Aiolos are both expressed to similar levels both in responses to SalOVA as well as alumOVA. This is of interest, for following *in vitro* polarization of activated CD4 T cells using IL-4, there was increased expression of *Il2*, *Ifnγ*, and *Tnfα* and decreased expression of *Il4*, and *Il13* in Ikaros^−/−^ CD4 T cells compared with wild-type CD4 T cells [20]. Ikaros is also important for IL-10 expression in T cells that have undergone Th2 differentiation, and a decrease in IL-10 expression in Ikaros^−/−^ CD4 Th2 cells was observed compared with their wild-type counterparts [22]. Ikaros appears to participate in T cell differentiation and maturation in other ways, for it also represses IFN-γ [21], [22]. At a molecular level, in Th2 cells derived *in vitro* there is evidence for Ikaros binding sites clustered in the *Il5* promoter and within *Il4* regulatory regions, including conserved noncoding sequence 1 (CNS-1), the IL-4 intron enhancer (I-E) and the V_A_ enhancer [20], [62], [63]. The binding of Ikaros to the *Ifn*γ CNS-22 element observed in Th2 cells may reflect its role in silencing *Ifn*γ. By contrast, in Th1 cells Ikaros binds predominantly to CNS-1 and the *Il4* promoter, and to a lesser extent to the *Ifn*γ promoter region [20]. Thus, Ikaros appears to exert distinct effects on *Il4* and *Ifn*γ in the same cell type (Th1 and Th2 cells) [20]. Ikaros also binds to the *Il10* promoter and intronic regulatory regions [22]. A highly conserved Ikaros binding site in the proximal *Il10* promoter (−901 bp) is located within 40 bp of a known GATA-3 binding site [22]. In line with this, Ikaros has been found to interact with GATA-1 in erythroid cells [64]. In addition, Ikaros interacts with GATA-1 and GATA-2 in mast cells but in this case negatively regulates IL-4 expression [62]. It is plausible that Ikaros interacts with GATA-3 in a similar manner in CD4 Th2 cells. As Helios dimerizes with Ikaros, it has been proposed that it acts as a rate limiting factor for Ikaros functions, thereby controlling its intracellular, cytoplasmic or nucleic, localization [9], [24], [31]. Ikaros and Helios share the similar binding DNA sequence (GGGAAT) [24]. Thus, it was tempting to speculate that Helios, Ikaros and GATA-3 may be part of a macromolecular complex that regulates accessibility at the *Il4* locus in CD4 Th2 cells. Finally, to add to the complexity both Ikaros and Helios have been shown to be able to potentiate as well as to inhibit gene expression [9], [21], [24].

So why were Helios^−/−^ CD4 T cells not impaired in Th2 differentiation? Perhaps this is because its function is compensated for by other members of the Ikaros family. Consistent with this, it is striking that a more severe phenotype is manifested by the *Ikaros* dominant negative homozygotes as compared with the *Ikaros* null mice [65]. This suggests that Ikaros dominant negative dimerizes and interferes with the activity of other Ikaros-like factors in the hematopoietic system. In addition, even though Ikaros has been shown to be required *in vitro* in IL-4-directed Th2 responses [20], [22] this does not necessarily imply that it is critical for the induction of early Th2-features in CD4 T cells in Th2 responses *in vivo*. We and others have shown that molecules important *in vitro*, including IL-4, may not be required *in vivo* for induction of primary Th2-features in CD4 T cells [57]. Thus, given the diversity in homodimerization and heterodimerization of the Ikaros family members, a full dissection of Helios activity during differentiation of Th2 cells *in vivo* may be required to get a clear understanding of the functional redundancies among Ikaros family members. This may require the development of genetic models where Helios can be studied in combination with Ikaros deficiency.

Are alum-precipitated antigens appropriate to explore the role of Helios? Undoubtedly Helios expression is associated with CD4 T cells responding to this form of antigen. Nevertheless, it is plausible that other types of Th2 responses may be more suited to assess the role of Helios *in vivo*. For instance, expression of the IL-4 receptor alpha, which is required for both IL-4 and IL-13 signalling is not required on smooth muscle cells for the development of experimental allergic asthma [66]. On the other hand, signalling through IL-4Rα contributes to initiation of Th2 immunity and pulmonary pathology during *Nippostrongylus brasiliensis* infections [67]. It also confers resistance during acute *Schistosomiasis* [68]. Thus, studies of the response of Helios-deficient mice to Th2-dependent parasites might reveal a role for this transcription factor during infections.

Finally, it has been proposed that Helios is a specific marker that distinguishes thymus-derived Foxp3 Tregs from those that are peripherally induced [27]. Our results show that Helios is also induced in peripheral mature CD4 T cells that respond to Th2 antigens without upregulation of Foxp3. Even though Helios is expressed at high levels in naturally occurring Tregs, two groups failed to detect a role for this transcription factor in the development and function of Tregs in their respective Helios knock-out mice [27], [55]. Thus, although Helios is a molecule expressed in thymocytes, Tregs and Th2 cytokine-producing cells its role in the biology of these cells remains obscure.

## Materials and Methods

### Mice and Chimeras

Wild-type C57BL/6J mice were from HO Harlan OLAC Ltd. (Bicester, UK). The Helios mice have been described elsewhere [55]. This strain was maintained on a C57BL6 background by crossing Helios^+/−^ mice. OTII mice that are transgenic for αβ TCR specific for 323–339 OVA-peptide in the context of H-2 I-A^b^ (Charles River, Wilmington, MA) were crossed to CD45.1^+^ C57BL/6 congenic mice (The Jackson Laboratory, Bar Harbor, Maine, USA), or NF-κB1^−/−^ C57BL/6 mice [69], or Helios^+/−^ C57BL/6 mice. NF-κB1^−/−^ mice were a kind gift from Dr. J.H. Caamaño (University of Birmingham, UK). All animal procedures were carried out in strict accordance with local ethical approval from the University of Birmingham and the UK Home Office license (Project license 40/2904) as covered by the Animals (Scientific procedures) Act 1986.

Fetal liver progenitors were prepared from E14 or E15 mouse embryos derived from pregnant females of the Helios^+/−^OTII^+/−^ mouse colony. These cells were frozen in a FCS 10% DMSO solution while each embryo was genotyped by PCR for Helios deficiency as previously described [55] and OTII transgene with primers for mouse OTII TCR α-chain: forward: 5′- AAAGGGAGAAAAAGCTCTCC-3′, reverse: 5′- CCAGCTGCGTCCCATCAC-3′. Chimeras were constructed by cell transfer of about 20×10^6^ freshly defrosted foetal liver progenitor cells, resuspended in a 150 µl volume of PBS and injected into the tail vein of lethally-irradiated (2×4.5G) C57BL/6 mice. In studies of the role of Helios in OTII cells, C57BL/6 or CD45.1^+^ C57BL/6 congenic mice for experiments performed at 3 days or 7 days, respectively, received 10×10^6^ CFSE-labelled LN cells from Helios^+/+^OTII or Helios^−/−^OTII chimeras.

### T cell purification, adoptive transfer and immunization

CD4 T cells from lymph nodes (LN) of CD45.1^+^ OTII mice were purified using anti-CD4 MACS microbeads (Miltenyi Biotec, Bisley, UK), CFSE-labelled (Cambridge Bioscience, Cambridge, UK) and 2×10^6^ cells were injected i.v. into CD45.2^+^ C57BL/6 congenic mice. Mice were immunized the following day in both footpads with alum-precipitated ovalbumin as previously described [5], [44].

### Flow cytometry, T cell analysis and FACS-cell sorting

Popliteal LN were prepared as previously described [5], [46]. Antibodies against B220- PerCP-Cy5.5 (RA3-6B2), CD4-PerCP-Cy5.5 (RM4-5), CD45.1-PE (A20), CD45.2-PE (104), CD45.2-PE-Cy5.5 (104), CD44-APC (IM7), CD62L-PE (MEL-14) CD69-biotinylated (H1.2F3), CD138-PE (281-2), CXCR5-biotinylated (2G8), Fas-biotinylated (Jo2), GL7-FITC (GL7), PD-1-PE (J43) and streptavidin-APC were from PharMingen (BD Bioscience, Oxford, UK) or eBioscience (Hatfield, UK).

Populations or OTII single cells were sorted by flow cytometry (MoFlo, DakoCytomation, UK) and the purity was routinely >90%. Final analysis and graphical output were performed using FlowJo software (Treestar, Costa Mesa, CA).

### Ex vivo restimulation and intracellular cytokine and immunoglobulin staining

Various days after primary or secondary immunization popliteal or brachial LN cells were incubated at 10×10^6^ cells/ml with 10 µM free 323–339 OVA-peptide for 5 h prior to cytokine detection. Intracellular FACS staining was performed using Cytofix/cytoperm kit (Becton Dickinson, Oxford, UK) according to manufacturer's instructions. Anti- IL-4-APC (11B11) is from PharMingen.

Intracellular staining to detect antibody-producing plasma cells was performed using BD cytofix/cytoperm kit. Goat anti-IgG1-alexa633 and anti-IgG2a-FITC were from Molecular Probe (Invitrogen, Paisley UK) and Southern Biotech (Cambridge BioSscience, Cambridge, UK), respectively.

### Foxp3, GATA-3 and Helios detection by intracellular flow cytometry

Intracellular staining was performed with the Foxp3 staining buffer kit, according to the manufacturer's protocol (eBioscience, Hatfield, UK). Anti-Foxp3-e-Fluor 450 (FJK-16s), anti-GATA-3-e-Fluor 660 (TWAJ), anti-Helios-Pacific Blue (22F6) and anti-Helios-PE (22F6) [27] were from BioLegend (Cambridge, UK) or eBioscience (Hatfield, UK).

### Immunohistochemical analysis

For *in situ* study of immune responses 5 µm cryostat sections were taken from snap-frozen LN for immunohistology as described in [60]. After cutting the first sections, which were used for immunohistology, two 25 µm sections of LN were cut, placed in a polypropylene microfuge tube, and stored at −70°C. Sections were fixed in acetone at 4°C for 20 minutes and air dried. The staining to reveal IgG1, IgG2a have been described in [57], [58], [70].

### Real-time RT-PCR

Real-time semi-quantitative RT-PCR on population, or LN sections, was performed as previously described [5], [71]. Briefly, probes and primers were designed using Primer Express software (Applied Biosystems) and sequences are detailed in Table S1. When possible, duplex were performed with β2-microglobulin and a target gene. Probes for cytokines and transcription factors were detected via a 5′ label with FAM, while probe for β2-microglobulin was 5′ labeled with NED.

Single cells were collected in 384 well plates containing 2 µl of cell-to-signal lysis buffer (Ambion, UK). mRNA were specifically reverse-transcribed and the PCR performed by adding 5 µl of a mix containing the Multiplex enzyme RT-PCR master mix (Qiagen Multiplex Probe RT-PCR kit), primers and probes. Details of the strategy and efficiency of the duplex and triplex RT-PCR on single cells are shown in the Figure S1. Standard reaction conditions for the TaqMan RT-PCR or PCR were used on the ABI 7900.

### In vitro Th2 polarization

Total LN OTII cells were incubated at 5×106 cells/ml in 6 well plates with 1 mM free 323–339 OVA-peptide (Alta Bioscience, University of Birmingham, UK) in complete medium, plus IL-4 (10 ng/ml) and neutralizing anti-IL-12 (C17.8) (5 mg/ml) plus anti-IFN-γ (X.MG1.2) (5 mg/ml) for 6 days. Restimulation was performed with anti-CD3 (145-2C11) coated (5 mg/ml) plus soluble anti-CD28 (37.51) (1 mg/ml) for 5 h at 37°C. Cytokines were from PeproTech and antibodies from Insight Biotech.

### Statistics

Statistical analysis was performed using a two-tailed non-parametric Mann-Whitney test. Values of p<0.05 were considered significant and all the p-values are indicated on the figures.

## Supporting Information

Figure S1 **Technique and strategy for duplex and triplex RT-PCR at the single cell level and its validation.** Consecutive two-fold dilutions of the mRNA template were amplified individually for IL-4 and each transcription factor gene product. The RT-PCR for GATA-3 or Helios were set up to work in triplex with β2microglobulin and IL-4 primers and probes, while Ikaros, c-Maf or NF-κB1 were set up to work with primers and probes specific for β2microglobulin only. **A**) CT plotted versus the 2 fold successive dilutions of mRNA, showing a linear quantitative relationship between the amount of mRNA template and the CT. This shows that RT-PCR run as simplex (open diamonds) or triplex (close diamonds) give equivalent results. Equations describing the trend lines are shown in each graph. We paid particular attention on the slope for each trend line and only accepted a difference of 0.1 between simplex and triplex RT-PCR. The correlation coefficients are also shown and are all close to 1. Pictures show the intensity of fluorescence as a function of the numbers of PCR cycles using SDS software (AppliedBiosystems) for varying numbers of FACS-sorted cells (32, 16, 8, 4, 2 see brown or purple lines) and 5 different single cells (see blue or green lines). Relative quantification for gene expression in single cells was achieved by setting thresholds (horizontal red line) within the logarithmic phase of the PCR and determining the cycle number at which the fluorescence signal reached the threshold (Ct). This shows that, as expected, the CT increases as the number of cells decreases. There is one blue flat line on each picture that shows a well without template that is with no FACS-sorted cell. **B**) Similar as in (A) for primer/probes that were run in simplex (open diamonds) or duplex (grey diamonds) RT-PCR.
(PPT)

Table S1 **Sequences for the primers and probes used in this study as indicated in the materials and methods in the section real-time semi-quantitative RT-PCR.** Sequences are written from the 5′ to the 3′ terminus. ^a^ Assay-on-demand (Applied Biosystem). GT: Germline Transcripts.
(DOC)
