# Supplementary material for: Helios Is Associated with CD4 T Cells Differentiating to T Helper 2 and Follicular Helper T Cells In Vivo Independently of Foxp3 Expression
Source: PLoS One. 2011 Jun 3;6(6):e20731. doi: 10.1371/journal.pone.0020731 (PMC3108993; doi:10.1371/journal.pone.0020731)
Supplement: Figure S1 — Technique and strategy for duplex and triplex RT-PCR at the single cell level and its validation. Consecutive two-fold dilutions of the mRNA template were amplified individually for IL-4 and each transcription factor gene product. The RT-PCR for GATA-3 or Helios were set up to work in triplex with β2microglobulin and IL-4 primers and probes, while Ikaros, c-Maf or NF-κB1 were set up to work with primers and probes specific for β2microglobulin only. A) CT plotted versus the 2 fold successive dilutions of mRNA, showing a linear quantitative relationship between the amount of mRNA template and the CT. This shows that RT-PCR run as simplex (open diamonds) or triplex (close diamonds) give equivalent results. Equations describing the trend lines are shown in each graph. We paid particular attention on the slope for each trend line and only accepted a difference of 0.1 between simplex and triplex RT-PCR. The correlation coefficients are also shown and are all close to 1. Pictures show the intensity of fluorescence as a function of the numbers of PCR cycles using SDS software (AppliedBiosystems) for varying numbers of FACS-sorted cells (32, 16, 8, 4, 2 see brown or purple lines) and 5 different single cells (see blue or green lines). Relative quantification for gene expression in single cells was achieved by setting thresholds (horizontal red line) within the logarithmic phase of the PCR and determining the cycle number at which the fluorescence signal reached the threshold (Ct). This shows that, as expected, the CT increases as the number of cells decreases. There is one blue flat line on each picture that shows a well without template that is with no FACS-sorted cell. B) Similar as in (A) for primer/probes that were run in simplex (open diamonds) or duplex (grey diamonds) RT-PCR. (PPT) [file pone.0020731.s001.ppt]

## Slide 1
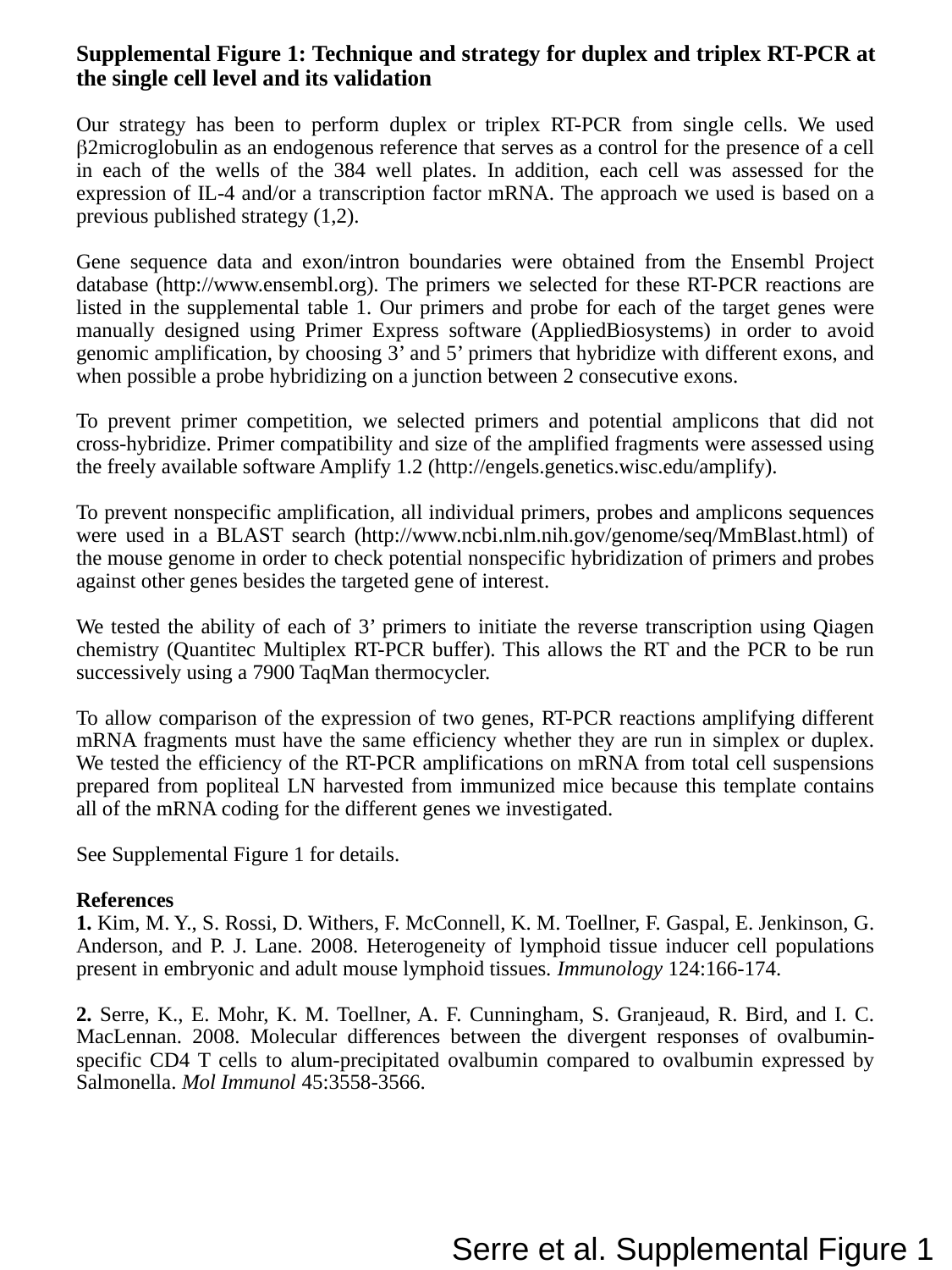

Supplemental Figure 1: Technique and strategy for duplex and triplex RT-PCR at the single cell level and its validation
Our strategy has been to perform duplex or triplex RT-PCR from single cells. We used β2microglobulin as an endogenous reference that serves as a control for the presence of a cell in each of the wells of the 384 well plates. In addition, each cell was assessed for the expression of IL-4 and/or a transcription factor mRNA. The approach we used is based on a previous published strategy (1,2).
Gene sequence data and exon/intron boundaries were obtained from the Ensembl Project database (http://www.ensembl.org). The primers we selected for these RT-PCR reactions are listed in the supplemental table 1. Our primers and probe for each of the target genes were manually designed using Primer Express software (AppliedBiosystems) in order to avoid genomic amplification, by choosing 3’ and 5’ primers that hybridize with different exons, and when possible a probe hybridizing on a junction between 2 consecutive exons.
To prevent primer competition, we selected primers and potential amplicons that did not cross-hybridize. Primer compatibility and size of the amplified fragments were assessed using the freely available software Amplify 1.2 (http://engels.genetics.wisc.edu/amplify).
To prevent nonspecific amplification, all individual primers, probes and amplicons sequences were used in a BLAST search (http://www.ncbi.nlm.nih.gov/genome/seq/MmBlast.html) of the mouse genome in order to check potential nonspecific hybridization of primers and probes against other genes besides the targeted gene of interest.
We tested the ability of each of 3’ primers to initiate the reverse transcription using Qiagen chemistry (Quantitec Multiplex RT-PCR buffer). This allows the RT and the PCR to be run successively using a 7900 TaqMan thermocycler.
To allow comparison of the expression of two genes, RT-PCR reactions amplifying different mRNA fragments must have the same efficiency whether they are run in simplex or duplex. We tested the efficiency of the RT-PCR amplifications on mRNA from total cell suspensions prepared from popliteal LN harvested from immunized mice because this template contains all of the mRNA coding for the different genes we investigated.
See Supplemental Figure 1 for details.
References
1. Kim, M. Y., S. Rossi, D. Withers, F. McConnell, K. M. Toellner, F. Gaspal, E. Jenkinson, G. Anderson, and P. J. Lane. 2008. Heterogeneity of lymphoid tissue inducer cell populations present in embryonic and adult mouse lymphoid tissues. Immunology 124:166-174.
2. Serre, K., E. Mohr, K. M. Toellner, A. F. Cunningham, S. Granjeaud, R. Bird, and I. C. MacLennan. 2008. Molecular differences between the divergent responses of ovalbumin-specific CD4 T cells to alum-precipitated ovalbumin compared to ovalbumin expressed by Salmonella. Mol Immunol 45:3558-3566.
Serre et al. Supplemental Figure 1

## Slide 2
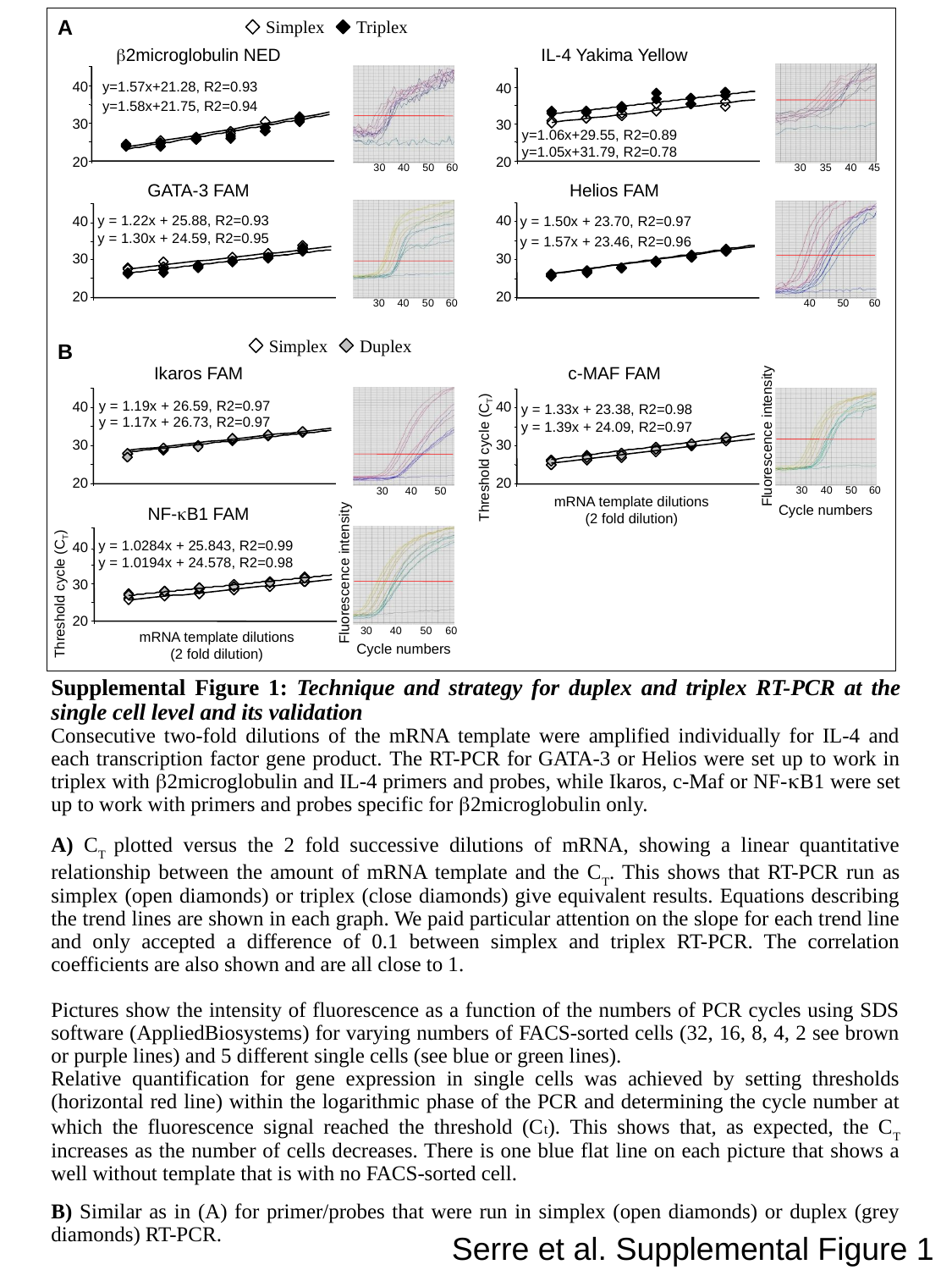

A
Simplex
Triplex
β2microglobulin NED
IL-4 Yakima Yellow
30
35
40
45
40
50
60
30
40
50
60
Fluorescence intensity
Cycle numbers
30
40
50
60
30
40
50
60
30
40
50
30
40
50
60
Fluorescence intensity
Cycle numbers
y=1.57x+21.28, R2=0.93
40
y=1.58x+21.75, R2=0.94
30
20
y = 1.22x + 25.88, R2=0.93
40
y = 1.30x + 24.59, R2=0.95
30
20
y = 1.19x + 26.59, R2=0.97
40
y = 1.17x + 26.73, R2=0.97
30
20
y = 1.0284x + 25.843, R2=0.99
40
y = 1.0194x + 24.578, R2=0.98
30
20
Threshold cycle (CT)
mRNA template dilutions (2 fold dilution)
40
30
y=1.06x+29.55, R2=0.89
y=1.05x+31.79, R2=0.78
20
40
y = 1.50x + 23.70, R2=0.97
y = 1.57x + 23.46, R2=0.96
30
20
40
y = 1.33x + 23.38, R2=0.98
y = 1.39x + 24.09, R2=0.97
30
20
Threshold cycle (CT)
mRNA template dilutions (2 fold dilution)
GATA-3 FAM
Helios FAM
B
Simplex
Duplex
Ikaros FAM
c-MAF FAM
NF-κB1 FAM
Supplemental Figure 1: Technique and strategy for duplex and triplex RT-PCR at the single cell level and its validation
Consecutive two-fold dilutions of the mRNA template were amplified individually for IL-4 and each transcription factor gene product. The RT-PCR for GATA-3 or Helios were set up to work in triplex with β2microglobulin and IL-4 primers and probes, while Ikaros, c-Maf or NF-κB1 were set up to work with primers and probes specific for β2microglobulin only.
A) CT plotted versus the 2 fold successive dilutions of mRNA, showing a linear quantitative relationship between the amount of mRNA template and the CT. This shows that RT-PCR run as simplex (open diamonds) or triplex (close diamonds) give equivalent results. Equations describing the trend lines are shown in each graph. We paid particular attention on the slope for each trend line and only accepted a difference of 0.1 between simplex and triplex RT-PCR. The correlation coefficients are also shown and are all close to 1.
Pictures show the intensity of fluorescence as a function of the numbers of PCR cycles using SDS software (AppliedBiosystems) for varying numbers of FACS-sorted cells (32, 16, 8, 4, 2 see brown or purple lines) and 5 different single cells (see blue or green lines).
Relative quantification for gene expression in single cells was achieved by setting thresholds (horizontal red line) within the logarithmic phase of the PCR and determining the cycle number at which the fluorescence signal reached the threshold (Ct). This shows that, as expected, the CT increases as the number of cells decreases. There is one blue flat line on each picture that shows a well without template that is with no FACS-sorted cell.
B) Similar as in (A) for primer/probes that were run in simplex (open diamonds) or duplex (grey diamonds) RT-PCR.
Serre et al. Supplemental Figure 1
