# Supplementary material for: Helios Is Associated with CD4 T Cells Differentiating to T Helper 2 and Follicular Helper T Cells In Vivo Independently of Foxp3 Expression
Source: PLoS One. 2011 Jun 3;6(6):e20731. doi: 10.1371/journal.pone.0020731 (PMC3108993; doi:10.1371/journal.pone.0020731)
Supplement: Table S1 — Sequences for the primers and probes used in this study as indicated in the materials and methods in the section real-time semi-quantitative RT-PCR. Sequences are written from the 5′ to the 3′ terminus. a Assay-on-demand (Applied Biosystem). GT: Germline Transcripts. (DOC) [file pone.0020731.s002.doc]

|  | **Primer Forward** | **Primer Reverse** | **Probe** |
| --- | --- | --- | --- |
| ***Aiolos*** | CATGAGATAGAAAACGTGGACAGTAGA | TTGCATAGCTGTAAGGCATTTCAC | ACAGAGATGAGAACATTATGAAGCCGGAGCC |
| ***β2 micro***  ***-globulin*** | CTGCAGAGTTAAGCATGCCAGTAT | ATCACATGTCTCGATCCCAGTAGA | CGAGCCCAAGACC |
| ***BCL6*** | CAGACGCACAGTGACAAACCA | ACTGCGCTCCACAAATGTTACA | CAGCCACAAGACTGTCCACACGGGT |
| ***c-Maf*** | ACCTCGGTCTTGCACTTTGC | TGCCTGGCTCTTATGGTTACTATTATT | CTCCGGATCCTTCGCGTGCGT |
| ***CXCR5*** | GCTCTGCACAAGATCAATTTCTACTG | CCGTGCAGGTGATGTGGAT | CCATCGTCCATGCTGTTCACGCC |
| ***Foxp3*** ^a^ | Mm00475156_m1 |  |  |
| ***GATA-3*** | CCACCCCATTACCACCTATCC | CACACACTCCCTGCCTTCTGT | TCGAGGCCCAAGGCACGATCC |
| ***Helios***  ***Exon4-5*** | ACGCTGTCACAACTATCTCCAGAA | GCTCTTGTTCCTTACAATCTTCCATA | ATGGAGGCTGCCGGGCAGGT |
| ***Helios***  ***Exon7*** | TCAAGGCTTTGGATGCTACCA | AAGGCCCTTATCTGTTCTCCTTCT | CTCTCTGAAGGACATCTATA |
| ***IFN-γ*** | TCTTCTTGGATATCTGGAGGAACTG | GAGATAATCTGGCTCTGCAGGATT | TTCATGTCACCATCCTT |
| ***IgG1 GT*** | CGAGAAGCCTGAGGAATGTGT | GGAGTTAGTTTGGGCAGCAGAT | TGGTTCTCTCAACCTGTAGTCCATGCCA |
| ***IgG2a GT*** | GGAACACTAAAGCTGCTGACACAT | AACCCTTGACCAGGCATCCT | AGCCCCATCGGTCTATCCACTGGC |
| ***IgE GT*** | AAGATGGCTTCGAATAAGAACAGTCT | CATGGAAGCAGTGCCTTTACAG | CTATCAGGAACCCTCAGCTCTACCCCTTAAAGC |
| ***Ikaros*** | CGCCCCAGGATCATTCTTG | CTTGACCCTCATCGACATCCAT | CGGCGCACAAATCCACATAACCTGAA |
| ***IL-2*** | AGCAATATCAGAGTAACTGTTGTAAAACTAAAG | CCACCACAGTTGCTGACTCATC | TCTGACAACACATTTGAGTGCCAATTCGA |
| *IL-4* | GATCATCGGCATTTTGAACGA | AGGACGTTTGGCACATCCAT | tgcatggcgtcccttctcctgtg |
| ***IL-5*** | AGAAATACATTGACCGCCAAAAAG | ACCAAGGAACTCTTGCAGGTAATC | CGTCCTCCGTCTCTCCTCGCCA |
| ***IL-13*** | TTGAGGAGCTGAGCAACATCAC | GCGGCCAGGTCCACACT | CAAGACCAGACTCCCCTGTGCAACG |
| ***IL-21*** | ACACCCAAAGAATTCCTAGAAAGACTAA | TGCATTCGTGAGCGTCTATAGTG | AGCATCTCTCCTAGAACACATAGGACCCGAAGAT |
| NF-κB1 | ACATGGTGGTTGGCTTTGC | CCCTAATACACGCCTCTGTCATC | AACCTGGGAATACTTCA |
| T-bet | ATGCCAGGGAACCGCTTATA | AACTTCCTGGCGCATCCA | CCCAGACTCCCCCAACACCGGA |

### **Supplemental Table 1:** Sequences for the primers and probes used in this study as indicated in the materials and methods in the section real-time semi-quantitative RT-PCR.

Sequences are written from the 5' to the 3' terminus. ^a^ Assay-on-demand (Applied Biosystem). GT: Germline Transcripts
